# Hybrid triboelectric-piezoelectric nanogenerator for long-term load monitoring in total knee replacements

**DOI:** 10.1088/1361-665X/ad3bfd

**Published:** 2024-04-18

**Authors:** Mahmood Chahari, Emre Salman, Milutin Stanacevic, Ryan Willing, Shahrzad Towfighian

**Affiliations:** 1 State University of New York at Binghamton, Binghamton, NY, United States of America; 2 Stony Brook University, Stony Brook, NY, United States of America; 3 University of Western Ontario, London, Ontario, Canada

**Keywords:** energy harvesting, triboelectric, piezoelectric composite, instrumented knee implant, silicone rubber, biomedical sensor

## Abstract

A self-powered and durable pressure sensor for large-scale pressure detection on the knee implant would be highly advantageous for designing long-lasting and reliable knee implants as well as obtaining information about knee function after the operation. The purpose of this study is to develop a robust energy harvester that can convert wide ranges of pressure to electricity to power a load sensor inside the knee implant. To efficiently convert loads to electricity, we design a cuboid-array-structured tribo-pizoelectric nanogenerator (TPENG) in vertical contact mode inside a knee implant package. The proposed TPENG is fabricated with aluminum and cuboid-patterned silicone rubber layers. Using the cuboid-patterned silicone rubber as a dielectric and aluminum as electrodes improves performance compared with previously reported self-powered sensors. The combination of 10$\,wt\%$ dopamine-modified BaTiO_3_ piezoelectric nanoparticles in the silicone rubber enhanced electrical stability and mechanical durability of the silicone rubber. To examine the output, the package-harvester assemblies are loaded into an MTS machine under different periodic loading. Under different cyclic loading, frequencies, and resistance loads, the harvester’s output performance is also theoretically studied and experimentally verified. The proposed cuboid-array-structured TPENG integrated into the knee implant package can generate approximately 15$\,\mu$W of apparent power under dynamic compressive loading of 2200 N magnitude. In addition, as a result of the TPENG’s materials being effectively optimized, it possesses remarkable mechanical durability and signal stability, functioning after more than 30 000 cycles under 2200 N load and producing about 300 V peak to peak. We have also presented a mathematical model and numerical results that closely capture experimental results. We have reported how the TPENG charge density varies with force. This study represents a significant advancement in a better understanding of harvesting mechanical energy for instrumented knee implants to detect a load imbalance or abnormal gait patterns.

## 1. Introduction

Knee osteoarthritis is a deteriorating joint disease that primarily affects 60-years and older men and women in the United States, and rates are rising quickly [1–5]. Total knee replacement (TKR) is one of the most effective interventions for the relief of chronic knee pain. A TKR is a surgical procedure that involves the removal of deteriorated bone and cartilage and their replacement with metal parts to reconstruct the surface of the joint between the femur and tibia [6]. Recent studies show that most TKR failures are related to instability, wear, and loosening that are often attributed to misalignment or overload related to patient activities [7]. Alignment and soft tissue balance have a significant role in determining the knee prosthesis lifespan as well as the patient’s postoperative mobility. To measure tibiofemoral joint forces, numerous implantable sensors have been presented. Implantable sensors enable self-monitoring systems to identify potentially hazardous loads and can inform prompt actions to protect the patient before the implant malfunctions. They can also be beneficial in post-operative joint monitoring, alerting patients and surgeons to any potential problems that could be addressed quickly, thus avoiding the need for a more complicated and costly procedure down the line [8, 9]. Therefore, the embedded pressure sensors can continuously supply information to orthopedic surgeons and detect potential problems with the joint before they worsen.

The optimal technique for data collection for *in*-*vivo* force measurement would enable the capture of information during and after surgery, over routine daily activities, without causing inconvenience or disturbance to the patient. For this purpose, a few recent studies have investigated smart knee implants with load-monitoring capabilities. Researchers have devised many sensing techniques requiring change of the tibial and bearing components to measure these loadings *in*-*vivo*. Heinlein *et al* [10] proposed a technique to measure the loading on the tibial component that embeds strain gauges into the stem. Marioli *et al* [11] proposed an electromagnetic generator that is incorporated into a knee implant capable of converting the mechanical energy produced by the movement of the knee into electrical energy to power a circuit within the implanted prosthesis. Piezoelectric energy harvesters utilize another mechanism to power wireless load sensors. Platt *et al* [12] studied the possibility of using piezoelectric ceramics inside TKRs to generate *in*-*vivo* electrical energy for diagnostic and monitoring purposes. Safaei *et al* [13] investigated the possibility of incorporating piezoelectric transducers into the polyethylene bearing of a TKR to act as a self-powered load sensor. However, piezoelectric energy harvesters are usually made of lead-based piezoelectric materials, such as PZT (lead zirconate titanate), which are not suitable for *in*-*vivo* biomedical applications due to their toxicity and brittleness caused by their ceramic crystal structure.

Triboelectric nanogenerators (TENG) have been widely employed as pressure, tactile, motion, or human-machine interface sensors because of the straightforward design, affordability, broad selection of materials, and the ability to directly characterize motion processes from the output signals [14, 15]. By taking advantage of the wide range of materials that possess triboelectric properties, it is possible to design triboelectric material pairs that are durable, ductile and efficient within the desired frequency range, thus eliminating the restrictions of piezoelectric devices such as brittleness and toxicity. TENGs can transform low-frequency mechanical energy into electrical energy through the combination of triboelectricity and electrostatic induction [16]. They have been used to create motion or shock sensors [17, 18] as well as load sensors. In our previous works [19, 20], we examined the performance of TENGs employing different dielectric materials (polydimethylsiloxane (PDMS), fluorinated ethylene propylene (FEP), and polytetrafluoroethylene (PTFE)) with different patterns for self-powered load sensors for TKR at 1000–2000 N of simulated gait loading. One of the major obstacles hindering the practical use of TENGs is the issue of mechanical damage, which occurred for the PDMS layer of previous TENG designs. When TENGs are damaged, worn particles can move to the surface of the other material involved in the process, leading to a decline in output performance, and reduced lifespan [21]. It is crucial to find a solution for achieving high durability performance, as this is a necessary step towards enabling the widespread and extensive implementation of TENGs into TKR implants.

Combining TENGs with a piezoelectric nanogenerator (PENG) (e.g. PZT, PMN-PT, PZN-PT) is a promising strategy to enhance energy harvesting capabilities [22, 23]. The hybrid tribo-pizoelectric nanogenerator (TPENG) creates electricity through the piezoelectric and triboelectric effects and their interactions. Piezoelectric materials that are typically used are often lead-based perovskites like PZT, PMN-PT, or PZN-PT. However, the environmental and health risks associated with lead, including the dangers of lead mining, manufacturing, usage, and disposal, are driving a shift towards lead-free solutions in the electronics industry. In this context, inorganic, lead-free piezoelectric substances like BaTiO_3_(BT) nanoparticles, which have high piezoelectric and dielectric properties, have been incorporated into various synthetic polymers to create a high-performance nanogenerator [24–26]. The BaTiO_3_ nanoparticle is a recognized piezoelectric and ferroelectric material. It shares similarities with lead zirconate titanate (PZT), and its piezoelectric harvesting characteristics have been extensively studied and described by numerous researchers [27, 28]. BaTiO_3_ piezoelectric nanoparticles are often chosen to be integrated into a soft polymer matrix to construct simple piezoelectric nanogenerators. The BaTiO_3_ has attracted remarkable interest owing to its non-polluting, straightforward preparation, and cost-effectiveness [29–31]. However, most energy harvesting papers on combined triboelectric-piezoelectric effects were focused on low ranges of pressure up to 20 KPa that are not suitable for biomedical implant applications [24]. We present characterization of a hybrid TPENG exposed to significantly high magnitude of pressure using a cuboid-array-structure for the goal of *in*-*vivo* measurement of loads on knee implants that can reach up to 0.93 MPa. The proposed sensor utilizes a cuboid-array-structured harvester using piezoelectric and triboelectric mechanisms. The output voltage signals generated by the device are a result of the alterations in deformation-induced electrostatic potential. Using the adhesive proteins found in mussels as a model, Poly(dopamine)(PDA) was used to modify BaTiO_3_ nanoparticles to enhance the compatibility between the inorganic filler with the silicone rubber matrix allowing better durability as well as higher electrical voltage stability. In fact, BaTiO_3_ stands out as a potential lead-free piezoceramic because of its remarkable piezoelectric constant ($d_{33}\gt$ 200 pC/N), its perovskite crystal structure that contributes to a high dielectric constant $(100-11\,\,000)$, and its biocompatibility [32, 33]. Consequently, the enhancements in durability and output efficiency make the proposed self-powered pressure sensor an ideal solution for long-term *in*-*vivo* measurements of knee implant loads.

The rest of the paper is organized as follows. In section 2, the working mechanism of the proposed cuboid-patterned hybrid TPENG is presented. In section 3, we used a lumped parameter model approach for developing the theoretical model. The characterization of the package-harvester assemblies and details of the related measurement results, as well as the verification of the theoretical model using experimental results are described in section 4. Finally, section 5 presents some brief conclusions.

## 2. Working principle

**Figure 1.**
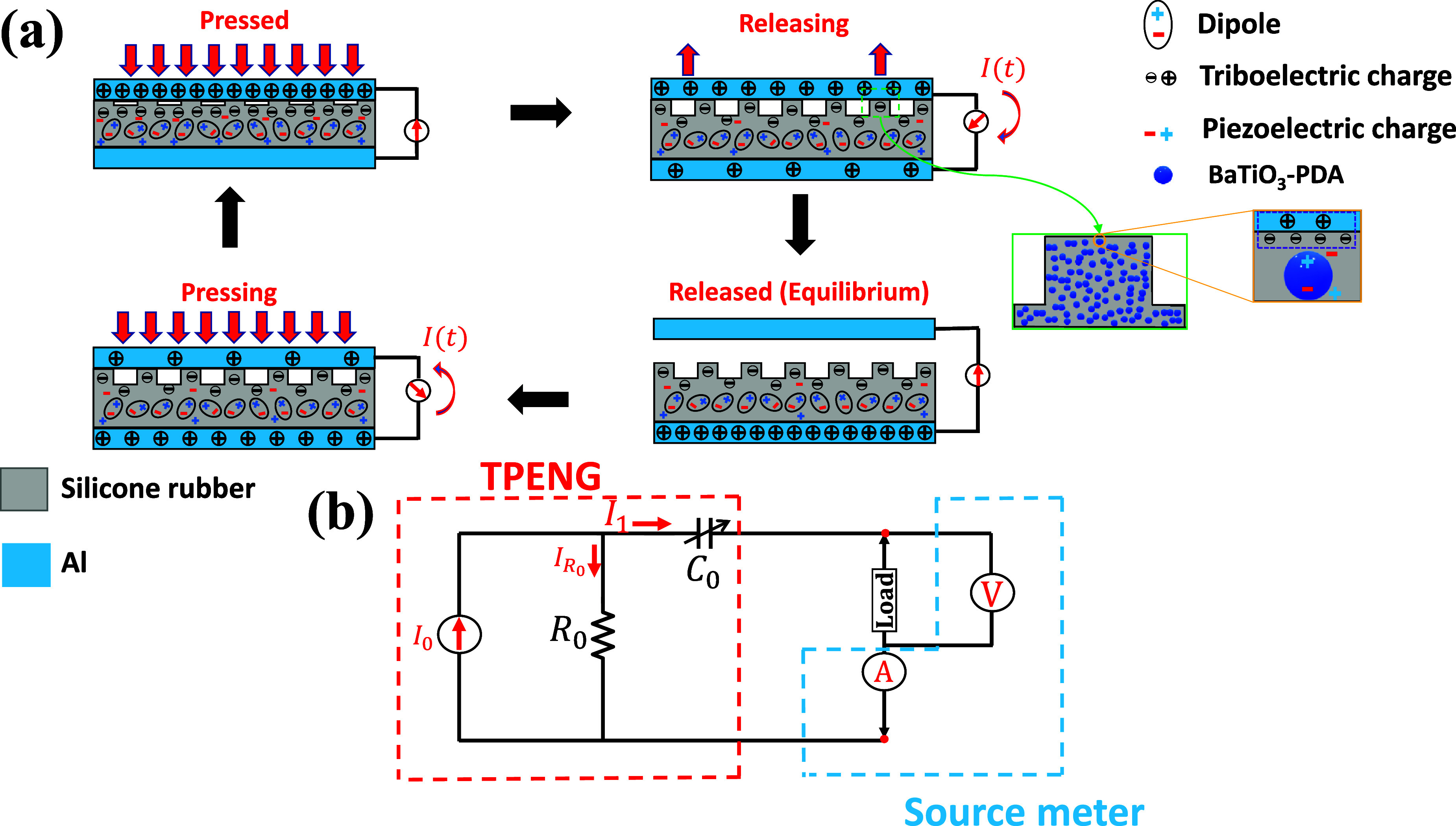
(a) Working mechanism of the contact-separation mode of proposed cuboid-patterned hybrid TPENG, (b) Schematic of the equivalent circuit model.

The working principle of the cuboid-array-structured BT-PDA/Silicone rubber TPENG is depicted in figure 1(a). The dielectric layer surface exhibits a regular arrangement of cuboid patterns, with BT-PDA nanoparticles evenly dispersed within the silicone rubber matrix. The composite layer of BT-PDA/Silicone rubber reported to yield significant output because of combined piezoelectric and triboelectric effects [24]. Because of its crystal structure characterized by pseudocubic symmetry, BT shows a strong piezoelectric response. Initially, the layers do not have any charges. Applying pressure results in the deformation of the cuboids within the silicone rubber along the vertical direction, generating an elastic force to counteract the distortion effect. Consequently, an electrostatic charge develops at the contact interface, resulting in a balanced distribution of positive charges on the upper aluminum electrode surface and negative charges on the silicone rubber surface. The upper aluminum electrode receives a positive charge, while the silicone rubber gets a negative charge because of their positions in the triboelectric series. Simultaneously, the BT-PDA nanoparticles embedded within the silicone rubber matrix experience compressive force, resulting in the generation of additional electric charge because of piezoelectric effect [34, 35]. Therefore, the bottom aluminum electrode becomes positively charged as a result of electrostatic induction. Removing the pressure causes the transfer of positive charges from the top to the bottom electrode, and also leads to the reduction piezoelectric charges, which creates the current until charges neutralize. The capacitor is currently charged. Once compressed again, the charged capacitor causes the current flow in the opposite direction. Increasing pressure leads to more friction between the BT-PDA/Silicone rubber and the aluminum electrode, boosting the compressive force on the BT nanoparticles (the triboelectric and piezoelectric charges are increased). This cycle then repeats periodically. Because of the combined effect of triboelectricity and piezoelectricity, the output performance is improved compared to pristine silicone rubber [24].

The cuboid-array-structured TPENGs operating in contact-separation mode can be characterized by a first-order equivalent circuit model according to these electrical characteristics: current *I*_0_, internal resistance *R*_0_, and internal variable capacitance *C*_0_ [36]. In this model, the resistor is linked in series with the capacitor and parallel with the current source. According to the governing equation for TPENGs operating in contact-separation mode, the peak output voltage of the pressure sensor is attributed to the applied pressure [24].

The proposed cuboid-patterned TPENG has a high internal impedance because of capacitance effect. This high-impedance TPENG can be regarded as a current source [37]. According to Kirchhoff’s Current Law (KCL) at the nodes, the equivalent circuit model of the TPENG can be developed as shown in figure 1(b).

## 3. Harvester package characteristics

**Figure 2.**
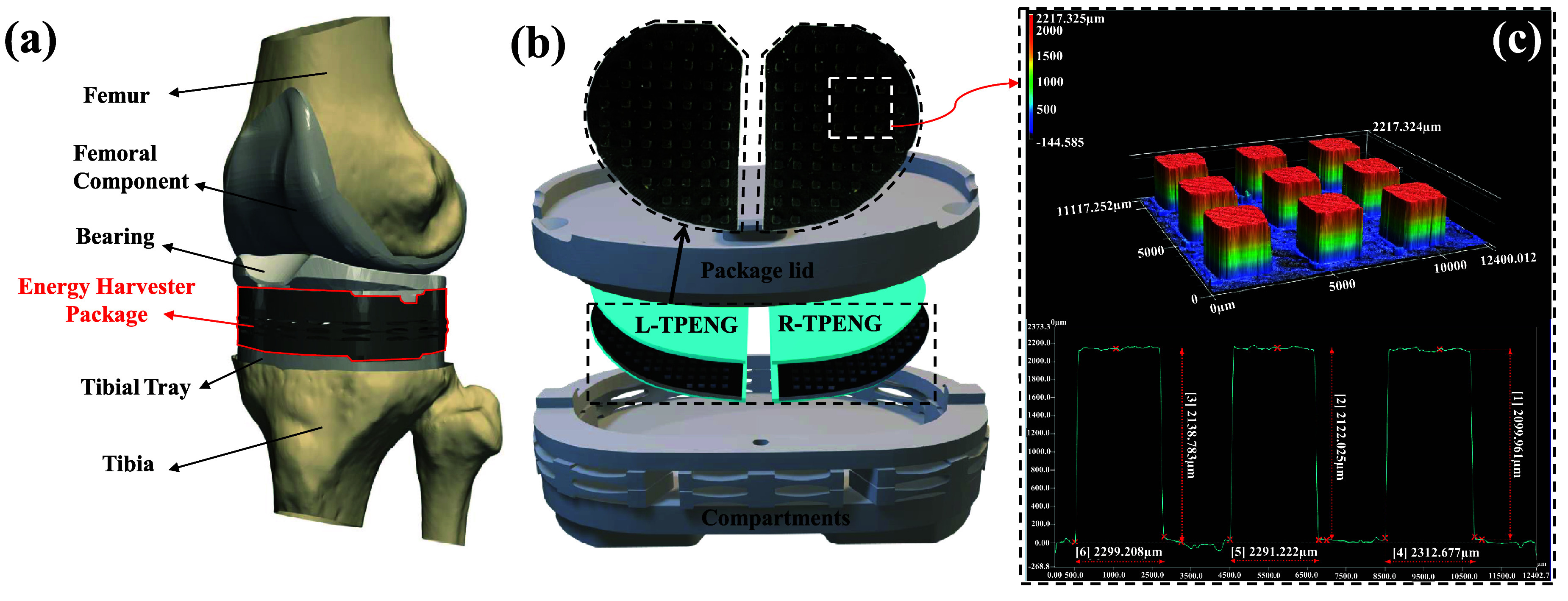
Self-powered load sensor for TKR: (a) Illustration of the Energy Harvester Package integrated into the joint replacement prosthesis, (b) Schematic of the components of an implanted package and the configuration of embedded TPENG inside the package, (c) The surface topography the harvester layer (the colorbar shows the measured values in $\mu \text m$).

An illustration of the harvester package and the embedded cuboid-patterned TPENG in the knee implant package are shown in figure 2. The surface morphology of the cuboid-patterned TPENG sample was analyzed and visualized using a 3D optical profiler (the KEYENCE VKX3000 model) at a magnification of 5x. Keyence Viewer surface metrology software was employed for 3D visualization of the captured images (shown in figure 2(c)). Additionally, 3D printed molds were quickly examined under an optical light microscope to identify any surface imperfections. For the experiments conducted in this study, we utilized displacement control on a universal compression testing machine (MTS 858 Mini Bionix II) to apply controlled forces to the harvester package. Based on the experimental measurements, a potential error of ±50 N was assumed for the force measurements conducted by the MTS.

**Figure 3.**
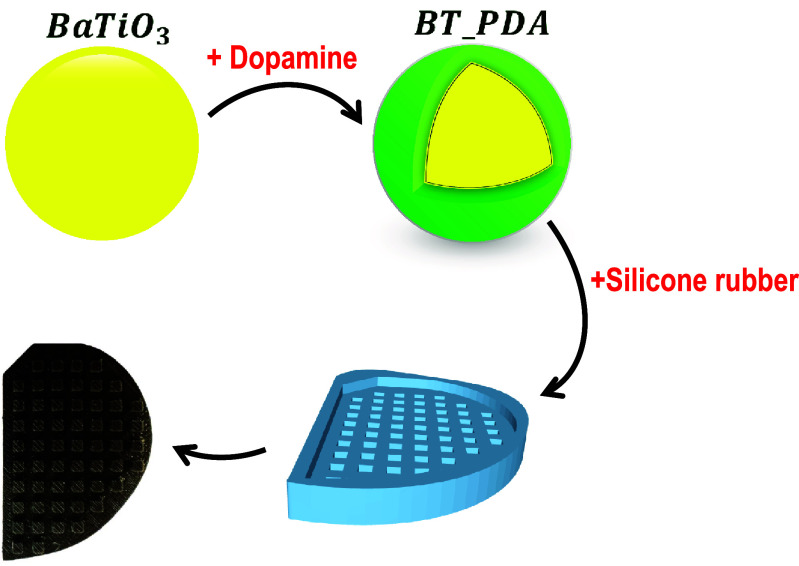
Schematic of the fabrication procedure to create cuboid-patterned silicone rubber layer using BT-PDA nanoparticles.

The sensitivity and measuring range of the cuboid-patterned TPENG were modified by regulating Young’s modulus of the silicone rubber material. Young’s modulus was enhanced by adding dopamine-coated BT nanoparticles and increasing their loading content in the silicone rubber matrix. To improve the interfacial interaction between the filler and matrix, the surface of the BT nanoparticles was modified using Poly(dopamine) (PDA) to create BT-PDA. The process for preparing BT-PDA nanoparticles and fabrication procedure for creating a cuboid-patterned silicone rubber layer using the dopamine-coated BT composite are shown in figure 3 (details can be found in the experimental section). Dopamine, a neurotransmitter found in the human body, has been utilized as a versatile biomolecule due to its adhesive properties and ability to form strong bonds with various surfaces [38]. In this case, dopamine was used to modify the surface of BT nanoparticles. The modification involved depositing a thin layer of dopamine onto the surface of the nanoparticles, which can then form covalent bonds with the silicone rubber matrix.

## 4. Mathematical modeling

**Figure 4.**
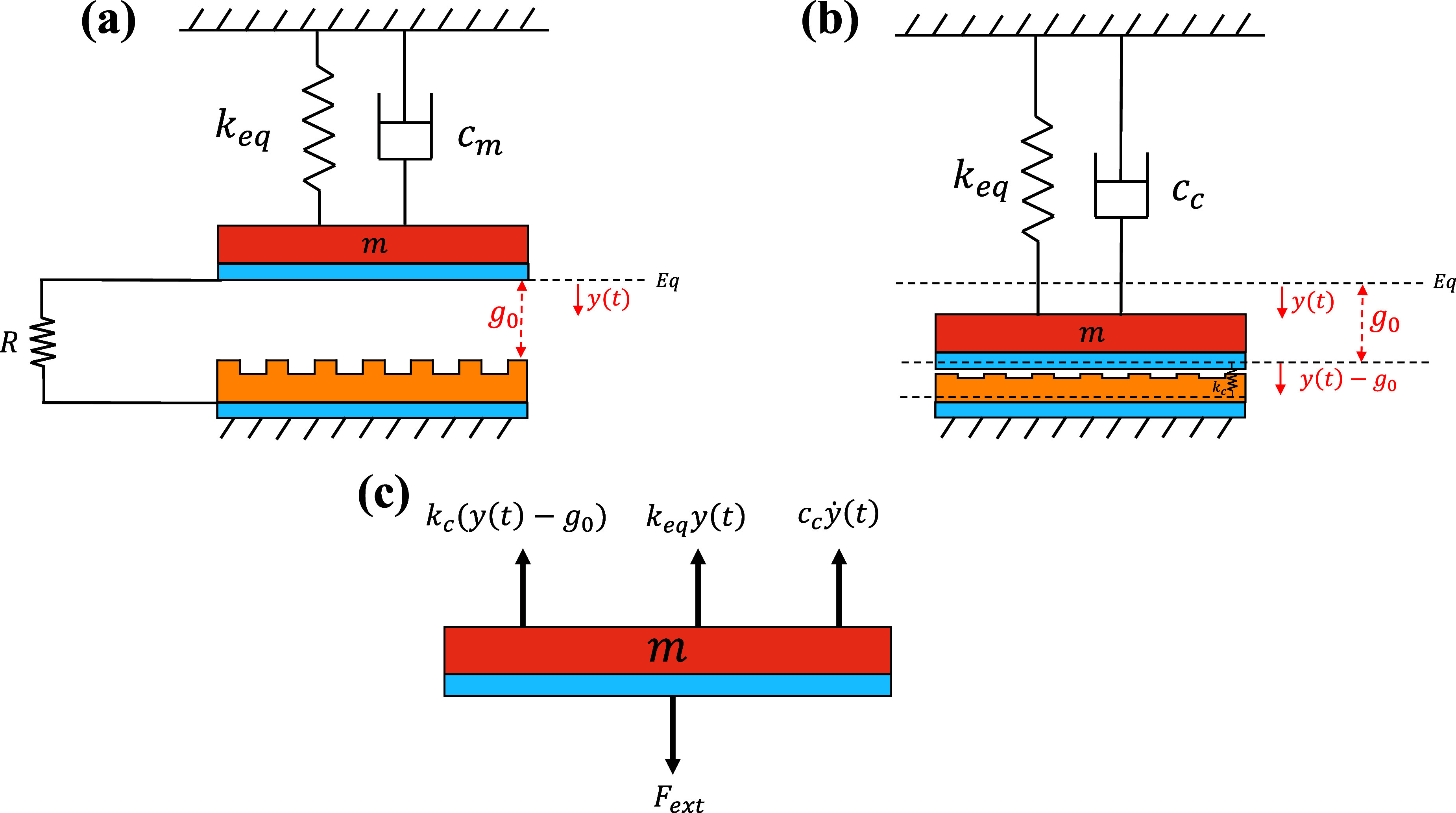
1DOF spring-mass-damper systems for the electromechanical coupling model of the TPENG package: (a) Pre-contact configuration; (b) During contact (c) Free body diagram during contact.

In this section, we present a mathematical model describing the output performance of the energy harvester package under cyclic loads. The model’s configuration consists of cuboid-patterned silicone rubber affixed to the lower electrode and an electrode attached to the package lid situated above them. The lower electrode and dielectric layer are held stationary, while the upper electrode undergoes reciprocating motion, applying a compressive force that results in triboelectrification. A model with lumped parameters is utilized to simulate both the dynamic response of a single-degree-of-freedom (1DOF) harvester package and the associated electrical output signal, as shown in figure 4. When an external force is applied to the harvester package, it can undergo two types of motion: the contact and the non-contact modes. Figure 4(b) provides a schematic representation of the system during the moment of the upper electrode’s contact with the silicone rubber layer, whereas figure 4(c) illustrates the free body diagram of the mobile mass after coming in contact with the silicone rubber layer. The system becomes stiffer and more damped after the contact, with the addition of extra contact stiffness (*k_c_*) and contact damping (*c_c_*), as shown in figure 4(b). If the deformation is less than the gap, no contact will happen between the upper electrode and dielectric layer; while compressing beyond the gap, causes the layers to touch. The electromechanical coupling governing equations can be presented as follows [39, 40]:

\begin{equation*} \begin{cases} m\ddot{y}\left(t\right) + c_m\dot{y}\left(t\right) + k_{eq} y\left(t\right) - F_e = F_{ext}, \quad y\left(t\right) &lt; g_0,\\ m\ddot{y}\left(t\right) + c_c \dot{y}\left(t\right) + k_{eq} y\left(t\right) + k_c\left(y\left(t\right) - g_0\right) = F_{ext}, \quad y\left(t\right) \unicode{x2A7E} g_0\end{cases} \end{equation*}

\begin{equation*} \dot{q}\left(t\right) = -\frac{q\left(t\right)}{\varepsilon_0 S R}\left(\frac{T}{\varepsilon_r}+g_0-y\left(t\right)\right)+\frac{\sigma}{\varepsilon_0 R}\left(g_0-y\left(t\right)\right) \end{equation*}

where *m* represents the effective mass of the package that can be expressed as ($m = m_{Al}+m_\textrm{pack}$), *y*(*t*) stands for the relative displacement of the upper electrode with respect to the equilibrium position, *c_m_* and *c_c_* denote the damping coefficients before and after contact, respectively, *k*_*eq*_ is the equivalent stiffness of the package, *k_c_* represents the stiffness after contact, and *F*_*ext*_ is the applied force to the harvester package. The harvester’s electrodes act as parallel plates in a capacitor with an electrostatic force that can be defined by $F_e = \frac{q(t)^2}{2 \varepsilon_0 \varepsilon_r S}$, where *q*(*t*) is the generated electric charges on the electrodes, *ε*_0_ is vacuum permittivity, *S* represents the effective contacting area of cuboid-pattern surface, respectively. Furthermore, *R* represents the load resistance, *T* is the silicone rubber layer’s thickness, *σ* is the surface charge density, and *g*_0_ is the initial gap. *ε*_*r*_ is the dielectric constant of a mixture of silicon rubber and dopamine coated BaTiO_3_ piezoelectric nanoparticles ($\varepsilon_r = 5$) [41]. MCR-5010 LCR meter was employed to verify the dielectric constant of the fabricated layer. Certain parameters of the model, such as contact-related stiffness, damping, and charge density have been estimated according to the experimental data. Furthermore, the effective surface area during contact (S) is estimated based on the experimental data, and it is expected to be smaller than the harvester area (A), as the effective contact area should be the sum of the cuboids area involved in contact when contact occurs. The physical and geometrical parameters are provided in table 1.

**Table 1.** Physical properties.

Properties	Symbol	Value
Vacuum permittivity	*ε*_0_	$8.85 \times 10^{-12}$ F m^−1^
Estimated dielectric constant of silicone rubber	*ε*_*r*_	5 [41]
Charge density	*σ*	39-64 *µ* C m^−2^
Harvester area	*A*	11.8 cm^2^
Effective surface area during contact	*S*	10 cm^2^
Silicone rubber thickness	*T*	2.7 mm
Initial gap	*g*_0_	0.2 mm
Frequency	*f*	1 Hz–2 Hz
Maximum sinusoidal loads	*F*_*max*_	200 N–2200 N
External resistance load	*R*	1 KΩ–2000 MΩ
Effective mass of the package	*m*	40 g
Effective damping	*c*_*m*_	0.02
Contact damping	*c*_*c*_	$10\times c_{m}$
Effective package spring constant	*k*_*eq*_	2100 N mm^−1^
Contact stiffness	*k*_*c*_	$20\times k_{eq}$

We assumed that the axial force profile applied by MTS follows a half-sine wave signal ($F_{ext} = \frac{1}{2} F_\textrm{max}(\textrm{sin}(2 \pi f t)-|\textrm{sin}(2 \pi f t)|$). The harvester output is influenced by factors such as the amplitude of applied force, excitation frequency, geometric shape, and electrical features like internal capacitance, internal resistance, external load resistance, dielectric constant, and charge density. We identify some of these parameters by comparing the simulation results of the aforementioned equations and the experimental data.

## 5. Results and discussion

**Figure 5.**
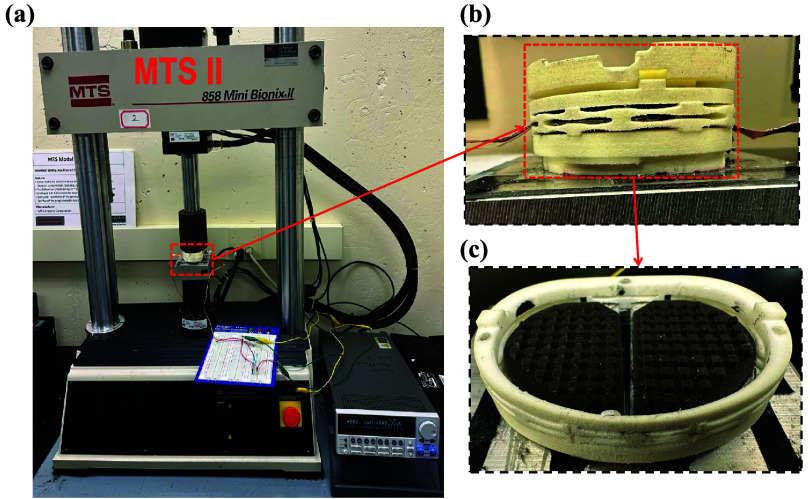
Illustration of the experimental setup for precise contact-separation TPENG measurements, (a) MTS 858 Mini Bionix II servo-hydraulic load frame (b) Schematic of the components of a harvester package prototype, (c) The configuration of embedded TPENG inside the harvester package.

In this section, we validate the theoretical model through experimental testing for the harvester package. Figure 5(a) illustrates the experimental setup for measurements, (b) side-view and (c) top-view image of the assembled harvester package. The mechanical and electrical output performance of the proposed self-powered TPENG pressure sensor within the implant package were investigated by conducting several tests on its electrical signals. An AC voltage signal was produced through cyclic contact and separation movements using a TPENG that operates in a vertical contact mode. The proposed cuboid-patterned TPENG components are attached within a mechanical spring-controlled housing to provide the required contact and separation. As in our previous study [20], we encapsulated the TPENG in a nylon package prototype. The geometry of the prototype was designed to fit a size 7 Triathlon tibial tray (Triathlon, Stryker, Kalamazoo, MI). The international standards organization regards that force waveform as the maximum for the typical axial forces found in TKR implants [42]. Human gait is reported to apply a range of forces between 2.5 to 3 times body weight at the knee joint [42, 43]. For a 75 kg person who under normal walking gait, the peak force is roughly 2200 N, which aligns well with peak values suggested in the literature of around 2.5–3 times body weight. Experiments were conducted for a 25% increase in maximum gait loads. Many studies on human motion energy harvesting have primarily focused on normal walking. Normal walking typically occurs within a frequency range of 0.5–2 Hz. Thus, we use the force up to 2.2 kN and frequency between 1-3 Hz for characterization of the harvester.

**Figure 6.**
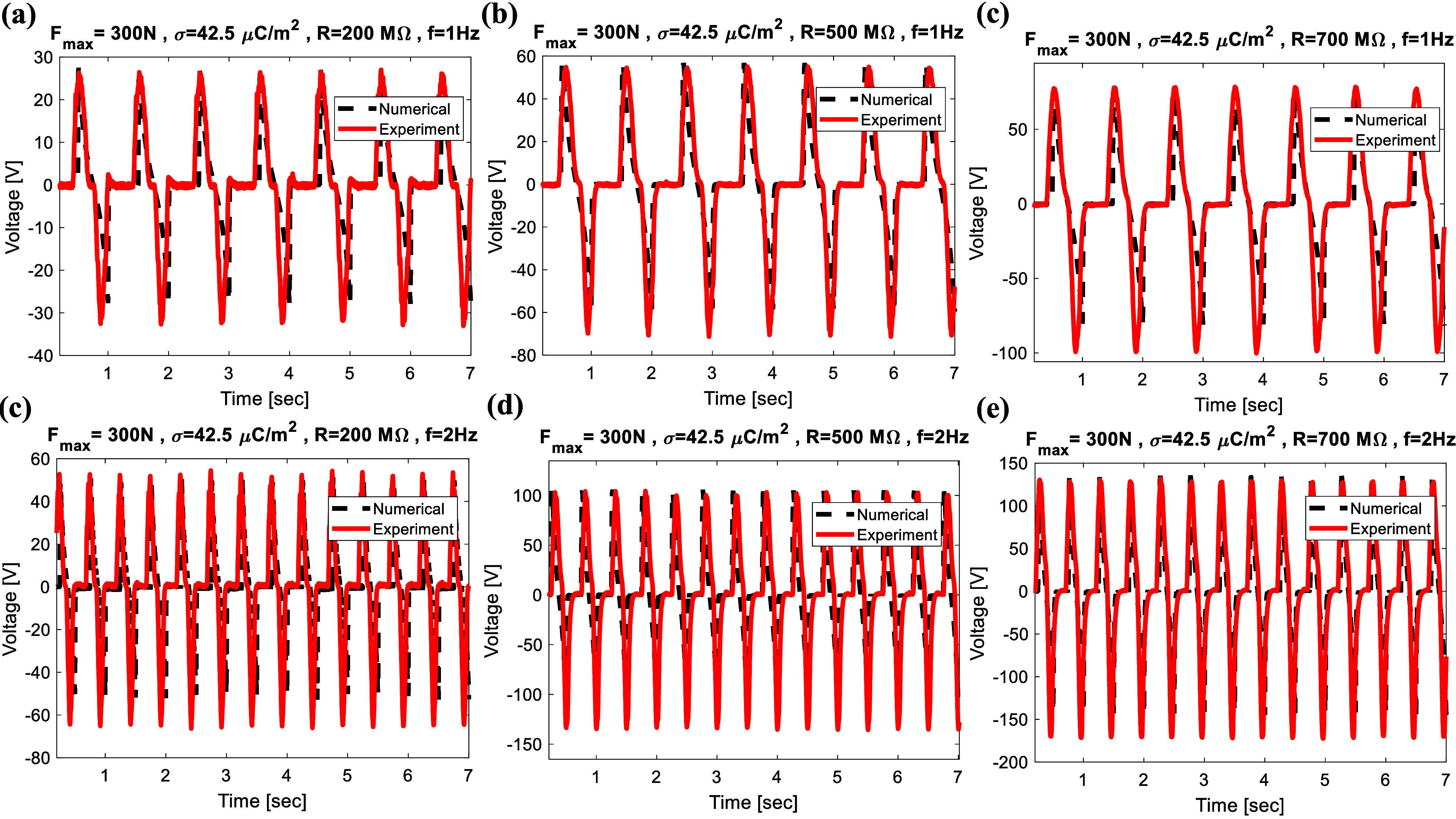
Comparison between the experimental and simulation output voltage for different load resistance and excitation frequency, under 300 N maximum applied force: (a)–(c) for load resistance of R = 200 MΩ, R = 500 MΩ, and R = 700 MΩ, respectively, at *f* = 1 Hz; (d)–(f) for load resistance of R = 200 MΩ, R = 500 MΩ, and R = 700 MΩ, respectively, at *f* = 2 Hz.

**Figure 7.**
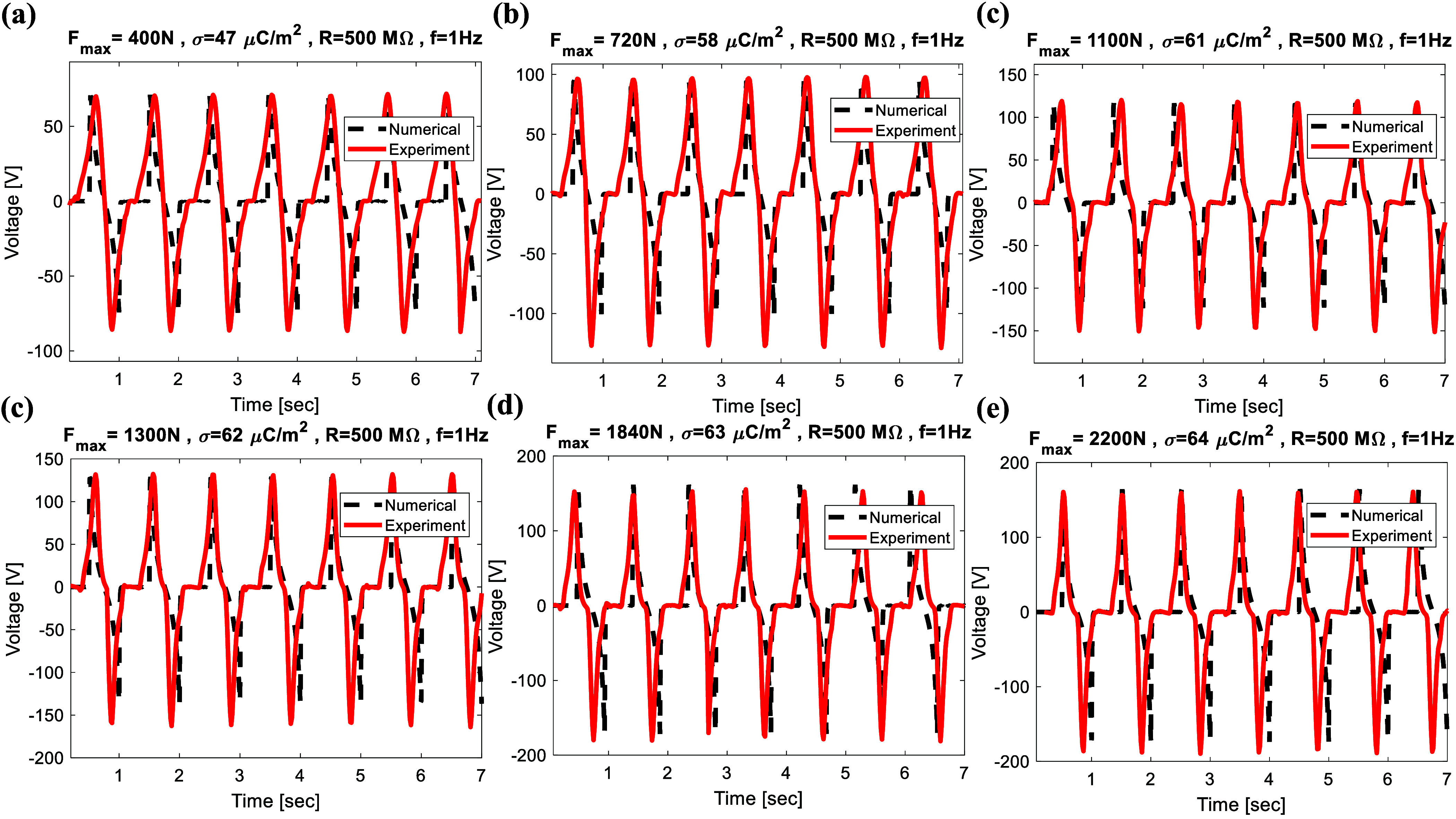
Comparison between the experimental and simulation output voltage for different maximum applied force: (a) $F_\textrm{max} = 400$ N, (b) $F_\textrm{max} = 720$ N, (c) $F_\textrm{max} = 1100$ N, (d) $F_\textrm{max} = 1300$ N, (e) $F_\textrm{max} = 1840$ N, (f) $F_\textrm{max} = 2200$ N, respectively, at *f* = 1 Hz.

To verify the accuracy of the electromechanical model presented in the previous section, a comparison between the experimental and simulation results has been carried out. To investigate the harvester’s dynamics through numerical methods, the material and structural parameters are listed in table 1. The coupled governing equations, as developed in the preceding section, were solved using MATLAB. As shown in figures 6 and 7, a comparison between the experimental and simulation output voltage for different external load resistance, excitation frequency, and applied forces was performed in order to verify the simulation results. Based on this comparison, the simulation results were in good agreement with the experimental data. The discrepancy between numerical results and experimental data may likely stem from the uniform charge density assumption in the numerical model, as well as factors such as variation in contact force during the cyclic loading test and nonlinear stiffness of the package, which is a visco-elastic material. These factors could lead to a time-dependent contact area and deviation between numerical and experimental data.

**Figure 8.**
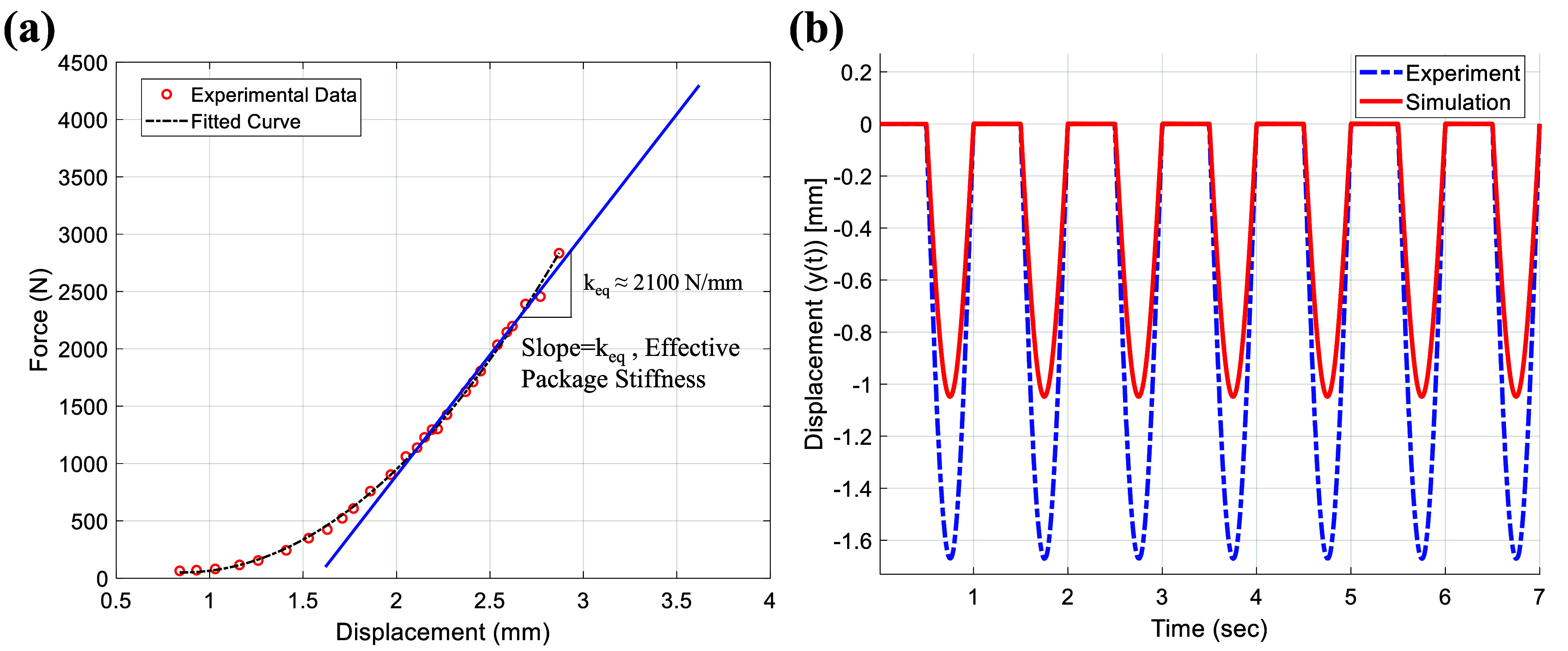
(a) Quasi-static force-displacement curve of the harvester package; (b) comparison between numerical and experimental results for the vertical displacement of the harvester package under cyclic loading of 2200 N.

The sinusoidal displacement excitations can be illustrated based on the recorded MTS head displacement profile. As shown in figure 8(a), the experimental force-displacement curve measured by the MTS test system with a quasi-static testing procedure, is used to estimate the effective harvester package stiffness ($k_{eq}\approx2100$ N mm^−1^). Figure 8(b) shows a comparison between the experimental results and the simulated axial displacement of the package based on the calculated effective stiffness of the package under cyclic loading of 2200 N. The observed deviation between the numerical simulation and experimental results can be attributed to several factors. First, the nonlinear stiffness of the package and tribo-pizoelectric layer (BT- PDA/Silicone rubber) can affect the displacement results. Second, it is important to consider the variation in stiffness of polymeric materials like nylon under different loading rates. As a result, the assumption of a constant effective stiffness in the simulation model may not accurately reflect the material’s response to varying loading conditions, potentially contributing to the observed discrepancies. Third, the experimental setup introduces additional variables and uncertainties, including imperfections in the flat surfaces and undesired tilt of the harvester package, which may have caused the presence of a very small gap between the MTS head and the package that influenced the observed discrepancy in displacement values.

**Figure 9.**
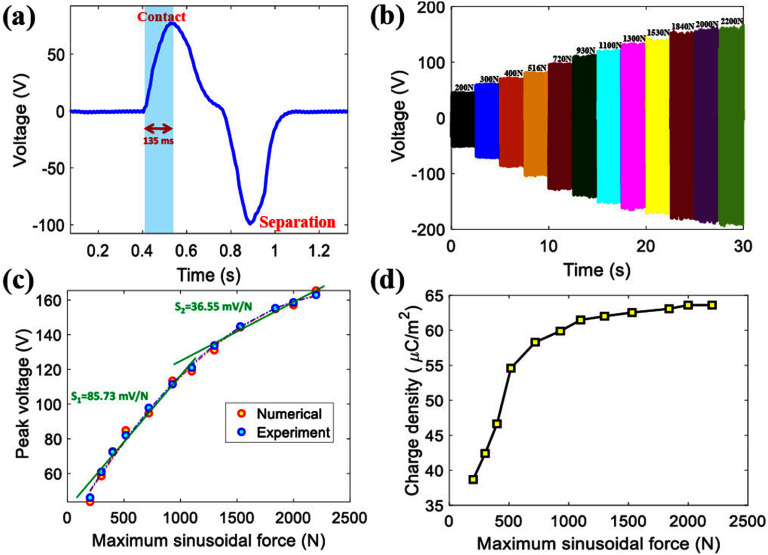
Characterizations of proposed self-powered cuboid-patterned TPENG as a load sensor: (a) The rise time of the output voltage signal, (b) The output voltage versus time signals for various applied loadings for load resistance of R = 500 MΩ (c) Comparison between the experimental and simulation peak output voltage under various maximum applied forces, (d) Experimentally obtained surface charge density with respect to the maximum applied forces.

The responsiveness of the self-powered TPENG pressure sensor under dynamic loading of 2200 N at 1 Hz is investigated. A magnified representation of the output voltage signal for a single contact-separation cycle is displayed in figure 9(a). The output voltage shows a rise time of around 135 ms, which is comparatively fast enough to detect changes in force as the person moves through different phases of the gait cycle. Figure 9(b) depicts the peak-to-peak output voltage signal vs time for the embedded cuboid-patterned TPENG under various applied forces (varies from 200 to 2200 N) at a constant frequency of 1 Hz. The output voltage was measured after hundreds of cycles during the stable operation period. Following each test run under a specific condition, the TPENG layers were left relaxed for a few minutes to enable the material to relax. This step is particularly crucial when one of the TPENG layers (in this case, BT- PDA/Silicone rubber) is a viscoelastic material [44]. By increasing the applied force from 200 to 2200 N, the output voltage increases because the stronger force results in greater overlap between the electron clouds of two triboelectric materials, leading to the release of more electrostatic charge density. In addition, the BT-PDA nanoparticles incorporated into the silicone rubber matrix undergo compression, leading to the generation of extra electric charge. This increase in charge generation ultimately leads to a higher output voltage. An important characteristic observed in figure 9(b) is the consistent and approximately symmetrical peak output voltage signal (around the zero voltage level) for all applied pressures. In TENG results reported in the literature, the non-symmetric and non-uniform voltage output is frequently observed. This is often attributed to issues with alignment and fluctuating contact conditions during the experiment [45]. As shown in figure 9(c), there is correlation between increasing applied forces and the output peak voltage. The surface charge density *σ* adopted in all simulations is derived from experimental results. The harvester’s output voltage across the load resistance is measured, and subsequently, the charge density value is adjusted in the numerical simulation to align the theoretical output peak voltage signal with the measured voltage. In addition, the experiment results demonstrate our self-powered sensor’s capability to detect force resolutions of 100 N for lower forces (less than 500 N) and approximately 200 N for higher forces. Furthermore, the proposed self-powered sensor demonstrates a sensitivity of 85.73 mV N^−1^ under forces lower than 1100 N and 36.55 mV N^−1^ under forces higher than 1100 N. Figure 9(d) illustrates the surface charge density with respect to the maximum applied forces. With the increase of applied load, the hybrid piezoelectric-triboelectric effect generates additional surface charges, resulting in an increase in surface charge density and output voltage.

**Figure 10.**
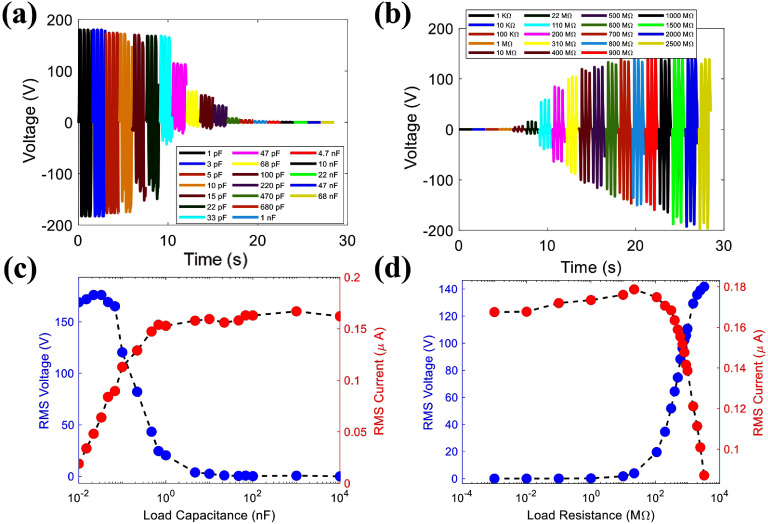
The voltage and current curves for various (a), (c) capacitance loads, and (b), (d) resistance loads.

**Figure 11.**
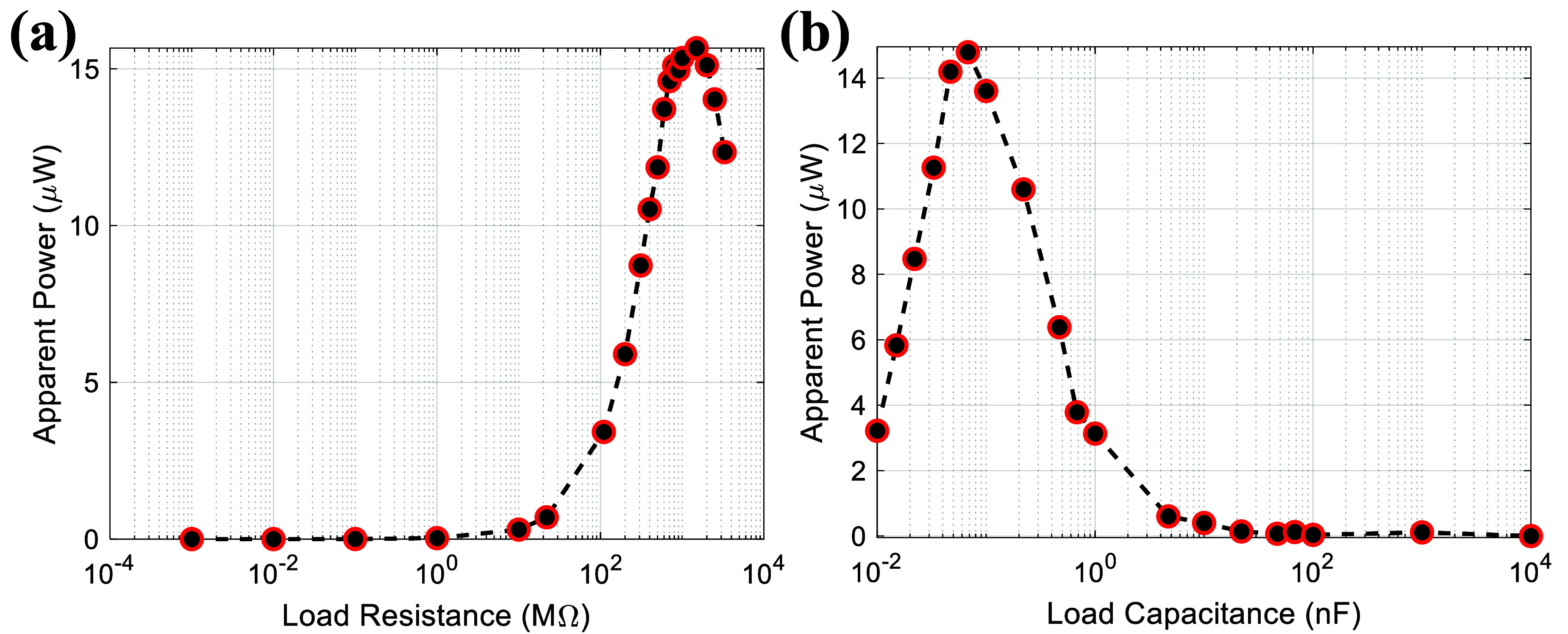
The relationship between apparent output power and external loads with a walking frequency of 1 Hz.

In engineering applications, a power-generating device such as TENG is connected to an external electrical load, and its power output can be affected by the external resistance load or unwanted capacitive loads. As shown in figure 10, the voltage and current characteristics of the embedded TPENG were measured while connected to a wide range of resistive and capacitive loads. The characterization was done for further development of a frontend electronic system that can benefit from our comprehensive study on the influence of external capacitor and resistor in the connected circuitry. This characterization will be used for the impedance matching of the frontend electronic system to maximize the energy transfer between the harvester and the power management circuit. As shown in figure 10, as the external capacitive load increases, the load voltage decreases, and the load current increases. On the other hand, as the external resistive load increases, the load voltage increases while the load current decreases. We employed the formula $P = {V_\textrm{RMS}}.{I_\textrm{RMS}}$ to obtain the apparent power curves depicted in figure 11. Through analysis of these curves, the maximum apparent power can be determined by identifying the specific external load at which the product of the RMS current and RMS voltage reaches its maximum value. The matching resistance and capacitance are one of fundamental factors used to assess the sensing capabilities of a self-powered sensor [46, 47]. Measuring the load voltage, current, and peak power is a specific approach to determining the matching resistance and capacitance. This method is commonly used to identify the appropriate values for the matching components in a circuit. As figure 11 illustrates, the matching resistance and capacitance are approximately in the range of 1500 MΩ and 68 pF, respectively. These tests were carried out to establish the quantity of apparent power that is dissipated in external loads connected to the harvester by subjecting the harvester package to cyclic loading at 1 Hz operating frequency. According to [48], the power consumption of a previous version of the frontend electronic system designed by our group is around 5.35 *µ*W. The proposed power management circuit in our previous study included impedance matching to the harvester impedance of 220 MΩ to maximize the power transfer. Using the presented harvester and reported frontend electronic system enables powering the system entirely from the harvester when the applied force reaches 2200 N (average dynamic load).

**Figure 12.**
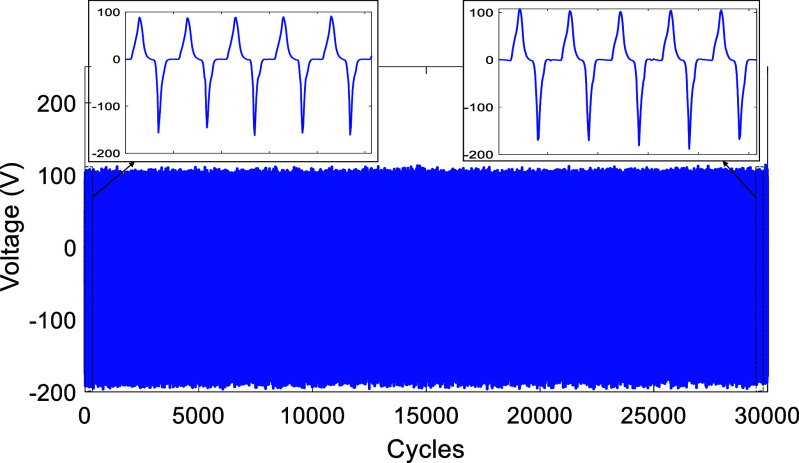
The long-term stability of cuboid-patterned TPENG embedded in Energy Harvester Package under dynamic sinusoidal loading of 2200 N at 1 Hz, demonstrating the stability of the TPENG.

Long-term stability and consistency are essential for TENGs in practical application. This means that harvesters need to maintain their performance over an extended time. To evaluate the harvester’s ability to maintain long-term stability, we conducted $30\,\,000$ cycles under identical conditions. As shown in figure 12, the proposed cuboid-patterned TPENG under dynamic sinusoidal loading maintained excellent stability, as depicted by the output voltage remaining constant. The durability of the proposed cuboid-patterned TPENG was found to be approximately three times greater than that of the previous work, with 30 000 cycles of stability compared to their reported 10 000 cycles [20], indicating a significant improvement of 200% in durability. The peak-to-peak voltage is nearly 270 V.

In general, the output performance of the TPENG harvester is significantly affected by the physical and chemical characteristics of the contact pair. In addition, the overall performance of the proposed TPENG is considerably influenced by various factors, including the magnitude of the applied contact pressure, operating frequency, and surface topography.

**Figure 13.**
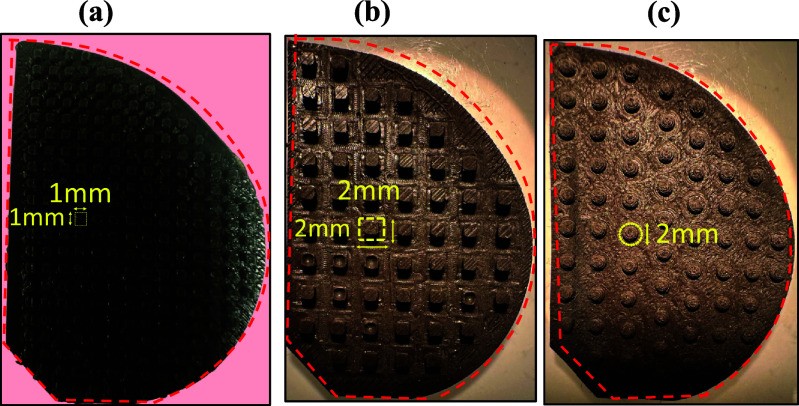
The schematics and dimensions of different harvester patterns, (a) 1 mm cuboids, (b) 2 mm cuboids, (c) 2 mm hemispheres.

**Figure 14.**
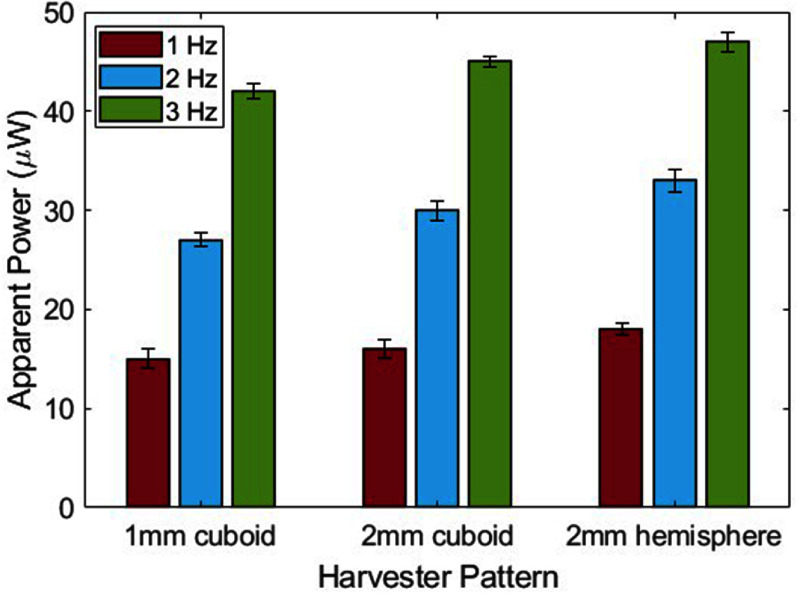
The apparent output powers for different harvester patterns under various frequencies.

Incorporating both macro/micro structures on the surface of silicone rubber can be an effective approach for improving the performance of triboelectric generators. Since different patterns change the area of the contacting surfaces directly affecting the charge density, we employed different patterns on the dielectric layer inside the energy harvester package (figure 13). Different patterns lead to changes in the contact area, charge transfer, and separation distance between the layers. By exploring various patterns, we can modify the energy conversion efficiency, power output, durability, and adaptability of the TPENG for specific applications and conditions. The experiments were conducted on various harvester patterns under a range of oscillating frequencies (1–3 Hz). The observed output powers were then determined for the different harvester patterns, including cuboid patterns with heights of 1 and 2 mm, and a hemisphere pattern with a diameter of 2 mm (figure 14). The results illustrated the maximum output power for various frequencies and patterns. As the frequency increased, the output power also increased because higher frequencies lead to a more charge flow. The accumulation and retention of triboelectric charges that are not neutralized during the shorter contact time related to higher frequency contacts as well as increase in current owing to moving charges in a shorter period are two causes of the increase in TPENG output with frequency. This means that as the contact and separation between the materials happens more frequently, there is a faster movement of the charges and a higher current is generated, leading to a higher output power of the TPENG.

Among the different patterns tested, the 1 mm hemisphere pattern yielded the highest apparent power of 45 *µ*W at 3 Hz operating frequency. The results show that a harvester package consisting of a half TPENG inside the package has the ability to generate an apparent power of around 15 *µ*W under normal walking conditions (1 Hz) and apparent power of around 45 *µ*W under normal running conditions (3 Hz). The parallel connection of left and right TPENG may enable doubling the power production as reported in our previous work [19]. According to previous studies, this power level is sufficient for powering a low-power sensor used to collect data *in*-*vivo* from TKR patients.

A comparison of this work with different energy harvesters embedded within smart knee implants for force sensing is presented in table 2. Several obstacles for knee implant applications include durability and lifetime, stability, output power, and biocompatibility. The table summarizes the performance of state-of-the-art devices on these categories. Two most common energy harvesting techniques are based on piezoelectric and triboelectric mechanisms. Most studies lack the information regarding the durability and stability of their embedded sensors within knee implants, which are crucial aspects for successful implementation. Many of these studies have focused primarily on reporting the output power over a limited number of cycles, often without comprehensive analysis or discussion on the longevity and robustness of the sensors. Since biocompatibility is an important aspect, we have also included this information in the table as well. While some piezoelectric ceramics have been reported to produce more power compared to triboelectric devices, they do not fit within the available thickness of tibial tray [49] and their brittleness make them prone to failure. Among triboelectric nanogenerators, our previously reported triboelectric harvesters used PDMS material, which showed output 12 % relative standard deviation (RSD%) of output variation over 10 000 cycles under the same loading [20]. This variation was reduced to 3 % (RSD%) over 30 000 in this study. These improvements enable having a more reliable self-powered load sensor with a longer lifetime.

**Table 2.** Performance of different energy harvesters embedded within smart knee implants for force sensing in the recent literature.

Type	Durability (Cycles)	Detection Range (kN)	Sensivity	Operating Frequency (Hz)	Voltage deviation (RSD%)	Max Output Voltage (V)	Apparent Output Power (*µ*W)	Biocompatibility
This work (TPENG)	$30\,\,000$	$0.1 \sim 2.2$	$36.55 \sim 85.72$ mV N^−1^	$1 \sim 3$	3%	160	$15 \sim 45$	$\checkmark$
Triboelectric [20]	$10\,\,000$	$0.05 \sim 2$	—	1	12%	130	19.81	$\checkmark$
Triboelectric [19]	—	$0.4 \sim 2$	—	$0.67 \sim 1$	—	60	$4 \sim 7$	$\checkmark$
Triboelectric [51]	—	$0.1 \sim 3$	—	1	—	30	8	$\checkmark$
Triboelectric [52]	1000	$0 \sim 0.05$	$99.56\ \mathrm{N}^{-1}$	1.2	—	4.5	—	✘
Piezoelectric [13]	—	$0.2 \sim 2.4$	—	$1.5 \sim 2$	—	2.3	3	✘
Piezoelectric [49]	—	$0 \sim 2.6$	—	1	—	40	27.6 mW	✘
Piezoelectric [53]	—	$0 \sim 0.2$	0.13 kPa^−1^	—	—	1	—	✘

Although we have carefully examined materials used and the durability of the self-powered triboelectric pressure sensor, there remains several challenges for future clinical application of the proposed harvester. Future developments will look into compactness, pre-clinical characterization, and imbalance output relationship. We will reduce the overall size of energy harvester to minimize the removal of excess bone. Pre-clinical testing will include inserting the harvester package into cadaver knees and finding the response to physiologically relevant conditions under joint motion simulators. In addition, we will determine the harvester output to load imbalance that enables early detection of aseptic loosening, a problem known in knee arthroplasty.

## 6. Conclusion

Consistent and effective monitoring of the loads is crucial to enhance the design of knee implants and identify early signs of implant misalignment. In summary, we have developed a highly durable energy harvester containing cuboid-array-structured TPENG for the measurement of a wide range of loads in knee implant packages. At the average walking force of 200–2200 N at 1 Hz, around 15 *µ*W of apparent power is produced, which is close to three times the power consumption of a prior harvester’s frontend electronic system in a Smart Knee Implant (5.35 *µ*W) [50]. Using hemisphere patterns and increasing the frequency can boost the output apparent power to around 45 *µ*W. As a result, this hybrid TPENG can generate practical electric power over tens of thousands of cycles, operating at the low frequencies and loads that are typical for a TKR application. The relative voltage change remained virtually unchanged after 30 000 dynamic loading cycles, demonstrating the pressure sensor’s outstanding mechanical and electrical stability. Our study on the proposed hybrid TPENG revealed minimal voltage deviation (RSD% below 3$\%$) compared to previous studies (earlier studies reported variations with RSD% over 12$\%$ for PDMS [20]). In addition, we have introduced a mathematical model and numerical results that are in close agreement with experimental results. With high sensitivity, a broad detection range, excellent stability, and durability, this harvester appears to be a promising option as a wireless pressure sensor for a knee implant application.

## 7. Experimental section

### 7.1. Fabrication of cuboid-patterned silicone rubber

BaTiO_3_ nanoparticles and dopamine were purchased from Sigma-Aldrich Co. LLC. The BaTiO_3_ had a density of 6.08 g ml^−1^ at 25 ^∘^C, and an average particle size of about 1 *µ*m. The modified cuboid-patterned silicone rubber was made first by dispersing 12 g of pristine BaTiO_3_ nanoparticles into 100 ml of ethanol and water mixture in a 1:1 mass ratio and stirred for 30 minutes. The solution was combined with 450 ml of deionized water and 1 g of dopamine hydrochloride. This mixture was stirred at 60 ^∘^C under electromagnetic stirring for 24 h, and then mixed by ultrasonic shaking for 30 min. The poly(dopamine) modified BT (BT-PDA) nanoparticles were achieved by filtration, then washed with deionized water and dried at 60 ^∘^C in a vacuum to prevent oxidation of the dopamine. The solution mixing method was used to disperse BT-PDA nanoparticles of 10 $wt\%$ concentration into the silicone rubber (Ecoflex 30) matrix. Afterward, the mixture was poured into a 3D-printed PLA patterned mold. Finally, the silicone rubber was peeled away from the mold to obtain the silicone rubber composite with 10 $wt\%$ BT-PDA.

### 7.2. Characterizations and measurements

The electrical outputs of the self-powered TPENG pressure sensor were measured at various forces and frequencies. Voltages and currents were recorded using an electrometer (Keithley 2636b System Electrometer). A 3D surface profiler with a super-depth-of-field (VK-X3000, Keyence, Japan) was used to examine the surface topography of the harvester layers. We also employed the MCR-5010 LCR meter to measure the capacitance and corresponding dielectric constant of the fabricated layer.

## Data Availability

All data that support the findings of this study are included within the article (and any supplementary files).
